# Studying exogenous extracellular vesicle biodistribution by *in vivo* fluorescence microscopy

**DOI:** 10.1242/dmm.050074

**Published:** 2023-08-01

**Authors:** Sien Yee Lau, Matthew Kang, Colin L. Hisey, Lawrence W. Chamley

**Affiliations:** ^1^ Department of Obstetrics and Gynaecology, University of Auckland, Auckland 1023, New Zealand; ^2^ Hub for Extracellular Vesicle Investigations, University of Auckland, Auckland 1023, New Zealand; ^3^ Department of Biomedical Engineering, The Ohio State University, Columbus, OH 43210, USA

**Keywords:** Extracellular vesicles, Exosomes, Microvesicles, Biodistribution, Fluorescence, Imaging, Labelling

## Abstract

Extracellular vesicles (EVs) are lipid-bound vesicles released from cells that play a crucial role in many physiological processes and pathological mechanisms. As such, there is great interest in their biodistribution. One currently accessible technology to study their fate *in vivo* involves fluorescent labelling of exogenous EVs followed by whole-animal imaging. Although this is not a new technology, its translation from studying the fate of whole cells to subcellular EVs requires adaptation of the labelling techniques, excess dye removal and a refined experimental design. In this Review, we detail the methods and considerations for using fluorescence *in vivo* and *ex vivo* imaging to study the biodistribution of exogenous EVs and their roles in physiology and disease biology.

## Teaser

**Summary:** This Review discusses how to optimise fluorescence imaging for studying the fate and biodistribution of exogenous extracellular vesicles, including fluorescent dyes, methods of labelling, excess dye removal and image acquisition.

## Introduction

Extracellular vesicles (EVs) are subcellular structures naturally released by all cells. They have phospholipid bi-layer membranes that encapsulate, protect and deliver diverse cargos, including lipids, proteins, RNA and DNA, around the body (Anand et al., 2019). Based on their size, EVs can be categorised into small EVs (<200 nm) and medium/large EVs (>200 nm), which encompass large apoptotic bodies (1-5 µm) (Théry et al., 2018). The biogenesis of EVs can result from a variety of processes, including the fusion of multivesicular bodies with the plasma membrane to form exosomes (a type of small EV), the outward budding of the plasma membrane, or the formation of apoptotic bodies released from a dying cell (reviewed in Mentkowski et al., 2018). Depending on their size and biogenesis, EVs may present with different external proteins – either anchored in the membrane, covalently attached to other proteins and lipids, or as part of the protein corona (Mentkowski et al., 2018) – that can play a role in their biodistribution.

EVs have been implicated in the modulation of physiological processes, such as immune modulation, as well as in diseases such as cancer and preeclampsia. EVs have shown promise as therapeutic treatments (Taraboletti et al., 2002; Kim et al., 2004; Brill et al., 2005; Hoshino et al., 2015; Wei et al., 2017; Han et al., 2019). Mesenchymal stem cell-derived EVs have angiogenic properties and can prevent long-term damage and improve outcomes following ischemic reperfusion injury in the kidney (Gatti et al., 2011) and heart (Lai et al., 2010). Furthermore, administration of mesenchymal stem cell EVs to mouse models of induced liver fibrosis improved the fibrotic phenotype (Li et al., 2012). As EVs can travel throughout the body via the circulatory and lymphatic systems, there is significant interest in where they ultimately distribute to and, from a therapeutic perspective, whether they can be used to target specific organs or tumours for drug delivery (Di Rocco et al., 2016; Lai et al., 2014).

Researchers have isolated EVs and exogenously applied them to rodent models to study their biodistribution. There are multiple ways in which biodistribution in general can be studied. Prior to the availability of *in vivo* imagers, biodistribution was studied by histological analysis of tissues (Attwood and Park, 1961), mRNA quantification (Bala et al., 2015), or flow cytometry of tissue lysates and blood (El-Assaad et al., 2014; Jaiswal et al., 2013). Modern tools for tracking exogenous EV biodistribution include bioluminescence labelling, radio-labelling or fluorescence labelling coupled with *in vivo* whole-animal imaging or *ex vivo* imaging of the organs of interest. There are several advantages and disadvantages to these labelling methods (summarised in Box 1), which have been extensively reviewed elsewhere (Cassidy and Radda, 2005; Gangadaran et al., 2018).

**Box 1. Advantages and disadvantages of commonly used labelling techniques to study extracellular vesicle (EV) biodistribution**

**Fluorescent labels**

[+] Accessible in most institutions

[+] Labels of different chemistries are widely available commercially and with robust staining protocols

[−] Many endogenous proteins are autofluorescent – careful selection of an appropriate label is important

**Bioluminescence**

[+] Bioluminescent signals are generated upon injection of a substrate, allowing for long-term (weeks to months) studies

[+] Low background bioluminescence in tissues and thus a strong signal-to-noise ratio, which allows for high sensitivity

[−] Requires transfection of EVs or the EV source with the substrate, which may not be possible or may result in non-uniformly transfected EVs, particularly in non-homogenous samples

**Radiolabelling**

[+] Highest sensitivity with very low background noise

[+] Large tissue penetration depth

[−] Requires specialised training, safety considerations and facilities

[−] Use of radiolabelling may be an additional concern in animal ethics applications

**Fig. 1.**
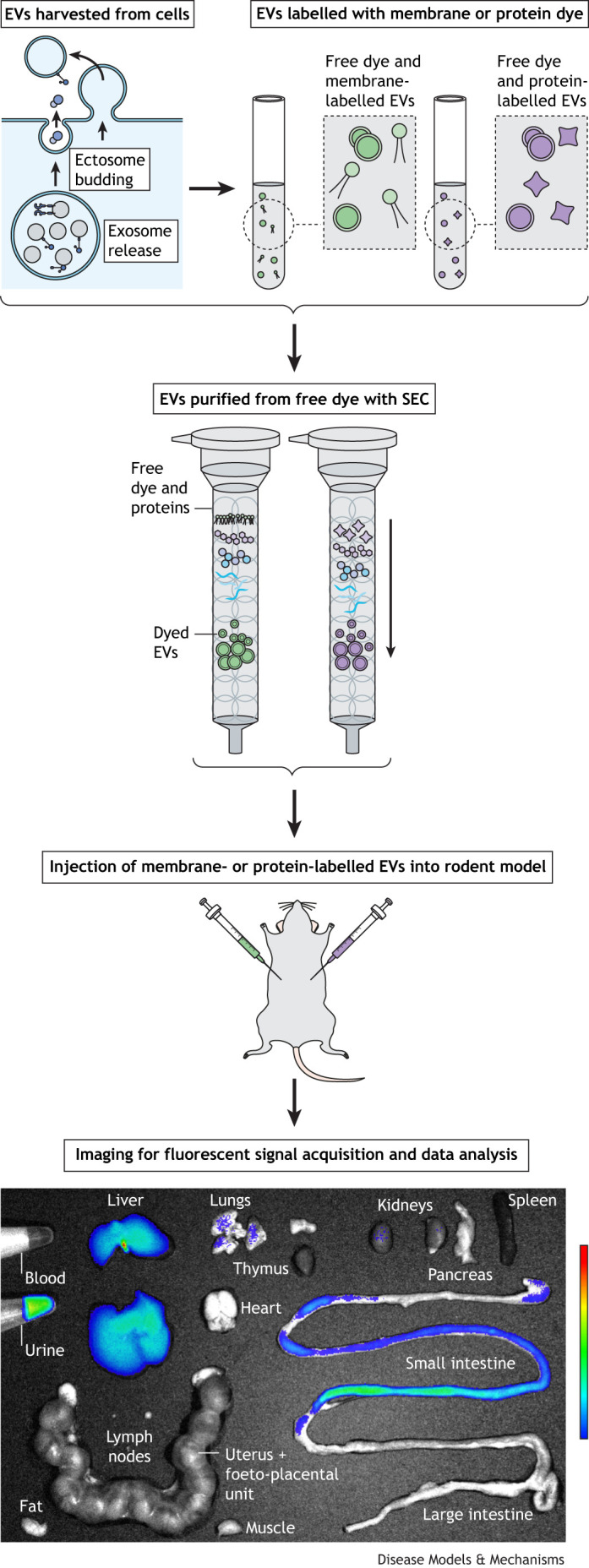
**A representative workflow for studying the biodistribution of exogenous EVs *in vivo*.** Harvested EVs from cells, such as placental syncytiotrophoblast, are labelled with either membrane- or protein-binding dye. Excess dye is removed using SEC or other purification methods (see also Fig. 2 for alternative methods), and the purified labelled EVs are injected intraperitoneally into mice. The organs of interest are then excised and imaged *ex vivo* using a fluorescence imager, and the outer border of each organ is carefully delineated to produce a region of interest. The imaging software then measures the photons emitted in each region of interest to quantify the fluorescent signal detected. The bottom panel shows an example from our own laboratory. In this experiment, we imaged murine organs *ex vivo* after the mice were injected with placental EVs that were labelled with Cyanine7 NHS ester. The organs were imaged using an AMI HTX system (Spectral Instruments Imaging). EV, extracellular vesicle; SEC, size-exclusion chromatography.

Fluorescence labelling remains the most commonly used and accessible technique, wherein EVs can be directly conjugated with a large range of commercially available fluorescent dyes and labels that are suitable for *in vivo* and *ex vivo* imaging (Table 1). The typical workflow for studying the biodistribution of fluorescently labelled, exogenously delivered EVs in a rodent model involves first harvesting EVs from cell culture or other sources and applying the fluorescent label to the EVs of interest. This may be by direct labelling methods in which the fluorescent dye is directly conjugated to isolated EVs, or the fluorescent dye can be applied to the live source cells/tissues in which the dye is packaged into EVs. Excess dye is removed, and the preparation is injected into the rodent model. The fluorescent signal is then captured using an imager, either in a whole animal *in vivo* or in organs of interest excised *ex vivo* (Fig. 1).

**Fig. 2.**
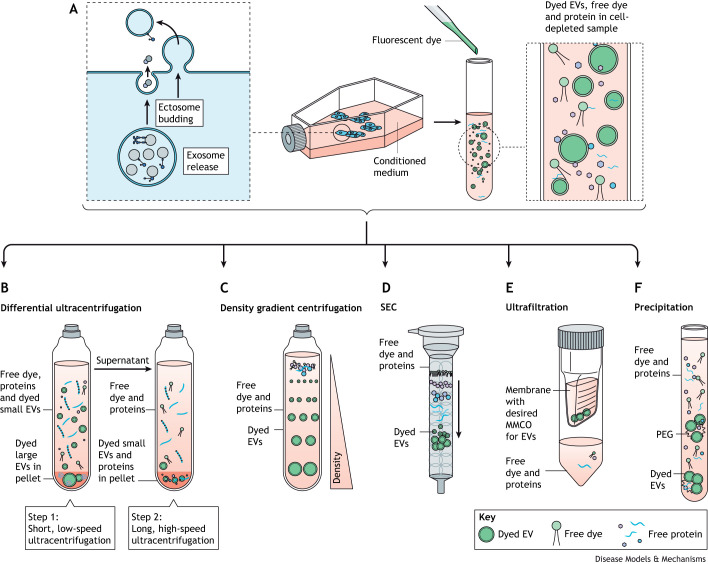
**Methods for the removal of excess dye.** (A) EVs can be harvested from conditioned cell culture medium or isolated from biological samples. After labelling with a fluorescent dye, the EV preparation contains a mix of labelled EVs and free dye. (B-E) Excess dye is typically removed by methods used in EV isolation processes, such as differential ultracentrifugation (B), density gradient ultracentrifugation (C), SEC (D) or ultrafiltration using membranes with appropriate MMCOs (E). (F) A less common method includes precipitation of the EV/dye complex. EV, extracellular vesicle; MMCO, molecular mass cut-off; PEG, polyethylene glycol; SEC, size-exclusion chromatography.

**Table 1.**
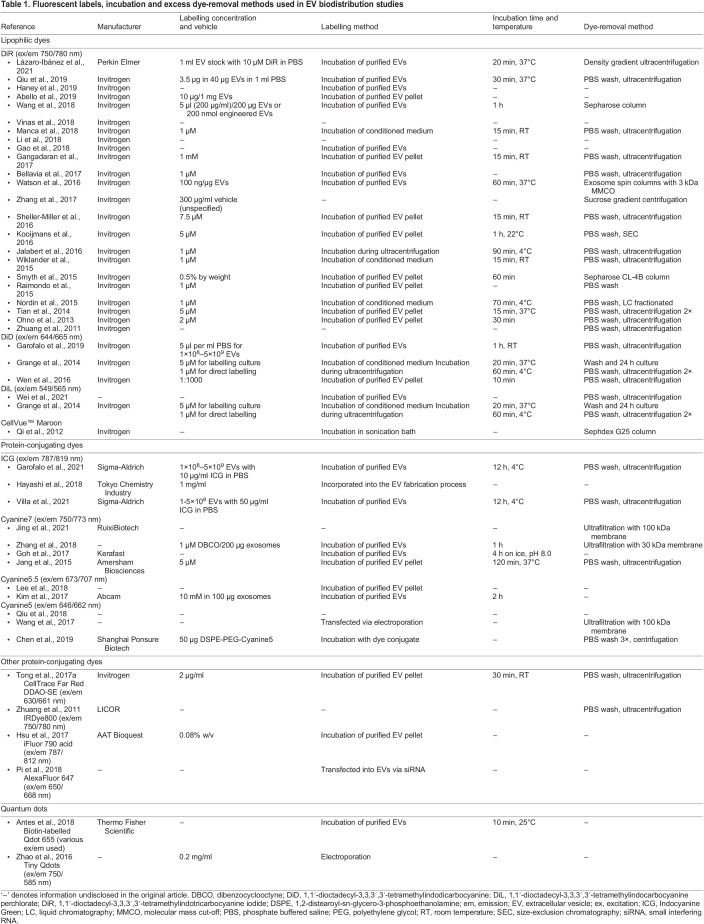
Fluorescent labels, incubation and excess dye-removal methods used in EV biodistribution studies

Unlike studying the biodistribution of cells, special considerations must be made when studying the biodistribution of EVs owing to their small size and lack of enzymes needed to activate some fluorescent dyes. Additionally, removal of excess dye requires techniques specific to EV processing. It is also clear that proteins on the surface of EVs are used to direct EVs to specific target organs and cells. Therefore, dyes that modify targeting proteins may modify the biodistribution of EVs (Nguyen et al., 2021). Despite these considerations and challenges, EVs have been an area of research with exponential growth in the past decade. Given the extensive technological progress in EV imaging, in this Review, we discuss the methodologies used in successful biodistribution studies of exogenously delivered EVs that employed fluorescence *in vivo* and *ex vivo* imaging (primarily in rodent models), and we highlight the considerations for future experimental design.

## Endogenous proteins, autofluorescence and signal absorption

The requirements for fluorescence *in vivo* imaging are different from those for fluorescence microscopy. *In vivo* imaging demands a much higher signal-to-noise ratio as the thickness of the tissue is in the range of centimetres, compared to micrometres for fluorescent microscopy (Crosignani et al., 2012). Owing to the need for deeper penetration, there are also much higher requirements for the power of the excitation light and the magnitude of the emission signal of the fluorophore (Crosignani et al., 2012) compared to those for standard fluorescence microscopy.

Some commercially available dyes bind to surface proteins; for example, N-hydroxysuccinimide (NHS) esters bind specifically to free amine groups (Lim et al., 2014). The number of dye molecules that can bind to the surface proteins of EVs will be limited to the number of freely available binding sites. When labelling with NHS esters, the availability of free amines on surface proteins for dye conjugation is limited owing to the small size of EVs compared to that of a larger target such as whole cells, which limits the strength of the fluorescent signal for biodistribution tracking. Similarly, EVs have a smaller lipid surface area for accommodating the penetration of lipophilic dyes, such as 1,1′-dioctadecyl-3,3,3′,3′-tetramethylindotricarbocyanine iodide (DiR), which represent the second core group of fluorescent dyes used for EV imaging (dye chemistries are discussed in more detail below). Given the fewer potential binding sites and entry points for fluorescent dyes, it is harder to detect a fluorescent signal from labelled EVs, and a higher signal-to-noise ratio is essential for achieving adequate sensitivity to study their biodistribution. Purpose-built fluorescent *in vivo* imagers employ ultra-cooled charge-coupled cameras that improve the sensitivity of detection and reduce background noise (discussed in detail later). However, other factors also contribute to the magnitude of the signal received, such as autofluorescence of and absorbance by endogenous proteins.

Endogenous proteins can emit photons when excited by light of certain wavelengths, a phenomenon described as tissue autofluorescence. For example, haemoglobin and structural components of human skin emit photons at 440 nm (Zheng et al., 2010) and 470 nm (Zeng et al., 1995), respectively. A range of diverse endogenous proteins, lipids and retinoids, as well as collagen and elastin found in connective tissues, emit photons with excitation and emission spectra that span 480-700 nm (reviewed in Croce and Bottiroli, 2014), creating background signals that must be overcome or avoided. Endogenous proteins may also absorb the photons emitted by the exogenously supplied fluorophores, preventing them from reaching the detecting camera. For instance, haemoglobin's absorbance should be considered when selecting the fluorescent labels for biodistribution studies in the blood. For *in vivo* fluorescence imaging, a ‘sweet spot’ exists in the near-infrared wavelengths (700-900 nm), which is a compromise to minimise absorbance by biological molecules while avoiding increasing absorbance by water beyond 900 nm (Zhang et al., 2012; Carr et al., 2018).

## Selection of fluorescent labels

Selection of an appropriate fluorescence label is an important consideration in the experimental design for *in vivo* imaging of exogenously injected EVs. Given the small size of the EVs, fluorescent labels that cause aggregation of labelled EVs, increase the size of the EVs after labelling or permit the aggregation of unbound label molecules can potentially confound the imaging and therefore the downstream interpretation of results. As we briefly introduced above, two main families of fluorescent dyes have been commonly used in the study of EV biodistribution to date: lipophilic dyes (Abello et al., 2019; Haney et al., 2019; Qiu et al., 2019; Wang et al., 2018; Lázaro-Ibánez et al., 2021; Qi et al., 2012; Wei et al., 2021) and protein-binding NHS ester dyes (Qiu et al., 2018; Garofalo et al., 2021; Jing et al., 2021; Zhang et al., 2018; Zhuang et al., 2011; Hayashi et al., 2018; Hsu et al., 2017; Pi et al., 2018).

Lipophilic dyes, such as DiR [excitation (ex)/emission (em) 750/780 nm], 1,1′-dioctadecyl-3,3,3′,3′-tetramethylindocarbocyanine perchlorate (DiL; ex/em 644/665 nm) and 1,1′-dioctadecyl-3,3,3′,3′-tetramethylindodicarbocyanine (DiD; ex/em 549/565 nm), insert into the phospholipid membrane of EVs. DiR is a widely used, commercially available fluorochrome that fluoresces brightly when bound to membranes and that differs from DiL and DiD mainly in the excitation and emission maxima. It is widely used, as its excitation and emission fall in the near-infrared spectrum, in which the background autofluorescence is lowest, giving a high signal-to-background ratio (see ‘Endogenous proteins, autofluorescence and signal absorption’ section). Although conjugation is reversible, DiR has been reported to only weakly fluoresce when unconjugated and suspended in aqueous solutions (Bruchez, 2015). Confirming this, studies that have injected free DiR intravenously into the tail vein of rodents as an experimental control group reported minimal background fluorescence (Li et al., 2018; Vinas et al., 2018; Gao et al., 2018; Raimondo et al., 2015). However, researchers have also noted that conjugation with DiR renders the EVs more prone to aggregation (Grange et al., 2014), which may alter the size and therefore the fate of the studied EVs. Accordingly, Dehghani et al. (2020) evaluated the size distribution of EVs labelled with various concentrations of the lipophilic dye PKH26 (ex/em 490/502 nm), another commonly used dye to label the lipid membrane of EVs. The authors reported significant increases in the size of EVs post-labelling, with the modal size of labelled EVs being twice that of unlabelled EVs (Dehghani et al., 2020). Lipophilic dyes have also been reported to self-aggregate to form micelles without binding to EVs, potentially confounding downstream analyses (Wallace et al., 2008; Lai et al., 2015; Kim et al., 2007). Given that cells may not be able to distinguish between dye aggregates and dye-conjugated EVs (Dehghani et al., 2020), we suggest that researchers determine the size of the EVs before and after labelling to ensure that the size has not been significantly altered by the labelling process.

Amine-reactive NHS ester dyes form stable amide bonds with free amine groups (Lim et al., 2014), thus labelling proteins on the surface of phospholipid membranes. Whether these dyes also passively diffuse through the EV membrane and label intravesicular proteins in a similar manner is an area of debate in the field, although Cyanine7 (ex/em 756/779 nm) and Cyanine7.5 (ex/em 788/808 nm) amine-reactive NHS esters have been successfully used for biodistribution studies (Goh et al., 2017; Zhang et al., 2018). One manufacturer (Lumiprobe) suggests incubating NHS esters with proteins in alkaline conditions at pH 8.4. However, proteins may be less stable in such conditions (Talley and Alexov, 2010), and consideration should be given to the adverse effect of pH on EV-surface proteins and potentially on the biodistribution of the EVs. NHS ester dyes are also less soluble than lipophilic dyes, and stock concentrations typically require resuspension in dimethyl sulfoxide (DMSO), which is toxic to cellular processes. The label–DMSO stock solution thus requires significant dilution prior to its addition to the EVs. Conversely, unlike lipophilic dyes, labelling with NHS esters has not been reported to cause EV aggregation.

Recent studies have also described chemical coupling of quantum dots to the outside of EV membranes as a fluorescent label (Antes et al., 2018; Zhao et al., 2016). Quantum dots are small particles (2-100 nm in size) made of semi-conducting material that can be excited to emit photons similar to fluorescent labels (Nguyen et al., 2019). Zhao et al. (2016) reported the use of engineered quantum dots with a size of 1.8 nm, which is smaller than the 15-20 nm size of commercially available quantum dots used in a subsequent study by another group (Antes et al., 2018). Researchers using quantum dots should consider their relative size to the EVs studied and the ratio of quantum dots bound to each EV, as this may substantially increase the EV–quantum dot aggregate size and potentially any size-based distribution pattern of the EVs of interest. In addition, the most commonly used quantum dots for medical research applications are made from cadmium-containing materials, such as cadmium selenide (Nguyen et al., 2019). Several studies have demonstrated that cadmium-containing quantum dots can be cytotoxic under certain experimental conditions (Nguyen et al., 2019), such as in oxidation, which leads to the release of cytotoxic cadmium ions into the surrounding tissue (Derfus et al., 2004).

It is important to note that we discussed the labelling methods described above in the context of exogenously delivered EVs. There is no evidence that the dyes we described, namely lipophilic DiR and amine-reactive NHS esters, show specificity for EVs. Therefore, if used for labelling endogenous EVs *in vivo*, there is a possibility that they may lead to undesired labelling of other lipids and proteins in the organism. The techniques employed for the labelling and study of biodistribution of endogenously released EVs *in vivo* have recently been reviewed elsewhere (reviewed in Verweij et al., 2021).

## Labelling methods

Fluorescent dyes can be used to label cells while in culture, prior to extrusion and collection of EVs. Donor-cell labelling typically has a lower efficiency than that of direct labelling of EVs, as it requires the incorporation of the fluorescent label into either the membrane or the cargo of the EV. The literature describes labelling of EVs at different stages of the isolation process, including direct labelling of purified EVs (Garofalo et al., 2019; Haney et al., 2019; Qiu et al., 2019), labelling of EVs in conditioned cell culture medium prior to their isolation (Manca et al., 2018; Wiklander et al., 2015) or labelling during EV isolation (Grange et al., 2014; Jalabert et al., 2016). Although adding the fluorescent dye during EV isolation shortens this time-intensive protocol, it might not provide the optimal labelling temperature and incubation times. Given the need for a high signal-to-noise ratio for imaging biodistribution *in vivo* or *ex vivo*, suboptimal labelling conditions may not achieve a sufficient number of bound fluorescent labels. Different methods of labelling may require different degrees of dye removal (discussed later in the Review) to ensure that free dye is not unknowingly injected with the sample and must be considered as part of the initial experimental design. Table 1 highlights the different labels and methods used in studies assessing EV biodistribution. Several researchers also report storage of EVs post-labelling at −80°C prior to use (Abello et al., 2019; Garofalo et al., 2019; Li et al., 2018) (Table 2).

**Table 2.**
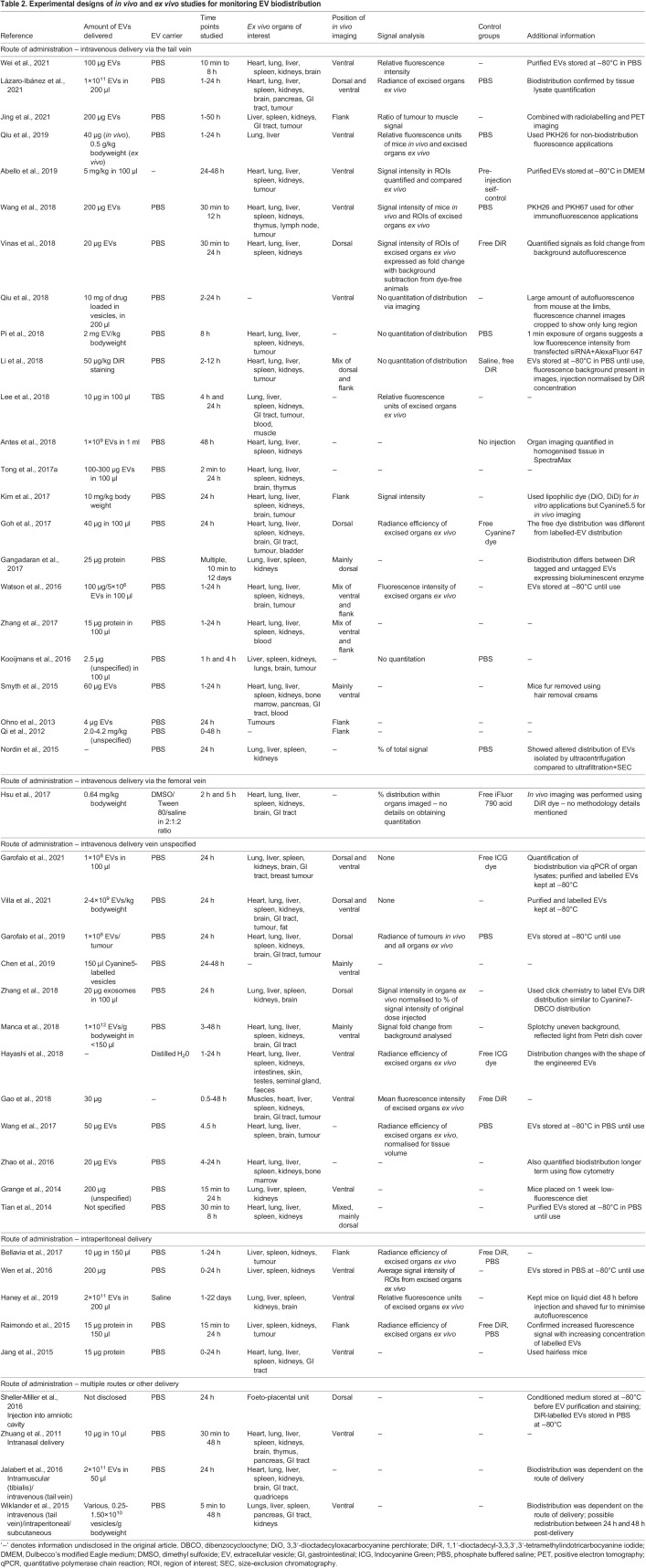
Experimental designs of *in vivo* and *ex vivo* studies for monitoring EV biodistribution

Although dye manufacturers supply protocols for labelling cell membranes, there is no suggested methodology for the labelling of EVs. This is reflected in the lack of standardisation in the literature, in which reported incubation times and temperatures range from 10 min (Antes et al., 2018; Wen et al., 2016) to 4 h (Goh et al., 2017), at 4°C (Goh et al., 2017; Jalabert et al., 2016; Nordin et al., 2015; Grange et al., 2014) to 37°C (Watson et al., 2016; Qiu et al., 2019). Lack of standardisation also extends to the labelling concentrations, which have been reported as microgram of dye per microgram of EV protein (Qiu et al., 2019), micromillilitre of dye per millilitre of carrier PBS without mention of the stock solution concentration (Garofalo et al., 2019), micromolar concentrations or the absolute amount of fluorescent dye per number of EVs. Inconsistencies in fluorescent label concentrations may lead to variation in the signal strength from the injected labelled EVs between replicate experiments. Although this variation is fine for identifying which organs EVs distribute to, it may make quantifications and comparisons between studies difficult. Reporting of standardised staining concentrations would enhance the comparability and reproducibility of studies published in the literature.

## Removal of excess dye

Successful removal of excess dye can be critical for accurately determining EV biodistribution, as any free dye administered to animals will result in unspecific labelling of tissues, confounding the EV biodistribution results. Several established dye-removal methods can be used independently or in combination. These methods are often the same as those used to isolate EVs, taking advantage of the differences in size, density or surface features between the EVs and excess dye. Unfortunately, every step in EV isolation or excess dye removal incurs significant losses in EV yield (Tsai et al., 2021), and the most appropriate method may differ depending on the source of the EVs (see Fig. 2 and Box 2).

**Box 2. Case study: biodistribution of EVs released from the placenta in patients with antiphospholipid antibodies**

The human placenta releases EVs into the maternal circulation throughout pregnancy as a form of maternal–foetal communication. These EVs are an important aspect of the maternal adaptation to pregnancy. The ‘message’ or the content of the EVs may change with maternal conditions that can affect placental health, such as the presence of antiphospholipid antibodies. Antiphospholipid antibodies are a key diagnostic marker for antiphospholipid syndrome, which is one of the leading causes of recurrent pregnancy loss. These autoantibodies affect the cargo of placental EVs (Tong et al., 2017b) and may also affect their distribution.

Given the limited availability of human tissues for research and the low recovery rate of EVs after removal of excess dye, our own group first sought to optimise the EV recovery to enable us to study placental EV biodistribution in patients with antiphospholipid syndrome. We compared the recovery rate of ultracentrifugation, ultrafiltration and size-exclusion chromatography after labelling placental EVs with Cyanine7 NHS esters. We showed that size-exclusion chromatography offers the best recovery of EVs post-labelling (Tsai et al., 2021). This pilot work enabled the collection of enough placental EVs post-labelling to perform a biodistribution study, in which we showed that the presence of antiphospholipid antibodies in the maternal circulation does not cause retargeting of placental EVs (Tsai et al., 2022).

The primary method for excess dye removal is ultracentrifugation, which separates EVs from excess dye by rotating the sample at high force (∼10,000-200,000 ***g***), creating a centrifugal field that causes the denser and more massive EVs to precipitate while excess dye remains in the supernatant. Density gradient ultracentrifugation uses a similar principle, but samples are loaded into density-specific layers of iodixanol or sucrose solutions, allowing the separation of components after they ultimately equilibrate in different layers based on their buoyant density (Veerman et al., 2021). This technique has recently garnered increased interest, as it may help to remove lipophilic dye aggregates, which can be the same size as the EVs.

Ultrafiltration is a size-based separation technique in which polyethersulfone, cellulose or other membranes with different pore sizes are used to separate EVs from the smaller excess dye. The sample solution is either driven directly into the porous membrane using a centrifuge, known as perpendicular or dead-end filtration, or repeatedly flowed tangentially over the membrane using a pump (Yang et al., 2020). In either case, the labelled EVs and anything larger than the specific membrane cut-off size remain above or on the membrane (retentate), while any smaller particles, such as excess dye molecules, pass through the membrane (permeate). For post-labelling clean-up processes, 500 kDa or lower-molecular mass cut-off membranes are typically used, and the choice of filtration system is largely based on the starting sample volume (Yang et al., 2020).

Alternatively, size-exclusion chromatography (SEC) uses a column packed with a matrix (e.g. Sepharose beads) of specific pore sizes to separate labelled EVs from free dye. The basic principle of SEC is that small molecules such as excess dye travel into and out of more pores within the matrix, whereas larger molecules cannot enter as many pores and travel more directly through the matrix. Thus, small molecules travel a much longer path through the column and are retained longer than the larger labelled EVs. The column effluent is normally collected in fractions, with the labelled EVs in earlier fractions than the excess dye.

These different dye-removal methods have their own advantages and disadvantages, and researchers must consider these when balancing between ensuring a clean sample free from excess dye, the appropriateness of the dye-removal technique for the EV type and source, as well as the loss of EVs incurred during the clean-up process.

## Selection of appropriate models and controls

The papers summarised in this Review use exclusively rodent models – mice and rats. It must be noted that other models have been used to study EV biodistribution *in vivo*, including transparent models such as *Caenorhabditis elegans* (Wang et al., 2014) and zebrafish embryos and larvae (Hyenne et al., 2019), which do not have the same challenges regarding autofluorescence (Androuin et al., 2021). Although the anatomy and physiology of *C. elegans* differ substantially from those of human, zebrafish contain a rudimentary circulatory system (Hyenne et al., 2019) necessary for studying biodistribution. By contrast, rodent models have higher similarity in anatomy and physiology to those of humans, with similar kidney and liver structures, which represent an advantage for studying EV biodistribution given that EVs distribute primarily to these organs (Kang et al., 2021). Additionally, rodent models allow the study of different routes of administration – intravenous, intramuscular, intraperitoneal, intranasal and via injection into the amniotic sac. Selection of the model organism should take into the account the suitability of the model to the organ system of interest (e.g. the presence and evolutionary conservation of the cardiovascular system), as well as the route of EV administration needed.

Additionally, selection of an appropriate control group is important to verify successful EV labelling and excess dye removal. Although the selection of control animals should be tailored specifically to the experimental question, any *in vivo* EV imaging experimental design should consider two fundamental controls – a dye-only control and an unlabelled EV (or PBS) injection control. Here, the dye-only control can inform the biodistribution of any excess dye that has not been removed in the EV labelling process (Garofalo et al., 2021; Hayashi et al., 2018). A PBS or saline injection control imaged at the same fluorescence spectra allows the identification of autofluorescence in the organs of interest, or lack thereof, allowing greater confidence in the data collection (Lázaro-Ibánez et al., 2021; Nordin et al., 2015; Bellavia et al., 2017). This could be further improved by the use of unlabelled EVs as a control, especially when imaging EVs that may have been engineered in a way that can affect their fluorescent properties.

## Fluorescence acquisition

*In vivo* imagers are a valuable tool in EV biodistribution studies, as they significantly reduce the work required for data acquisition compared to histological analyses and processing organ lysates. This allows for a larger number of experiments, increasing the statistical power. Importantly, *in vivo* imaging can be performed in a live, anaesthetised animal to track changes in biodistribution over time in the same animal.

Major *in vivo* imagers are engineered such that the light source and acquisition camera are positioned on top of the capture stage. Cooled charge-coupled devices reduce thermal noise. When using *in vivo* imagers, researchers should consider multiple factors to ensure accurate biodistribution analysis.

### Imaging parameters

Several settings, which are general to photography, can be adjusted to optimise the brightness of the fluorescence signal detected by *in vivo* imagers, including exposure time, excitation strength, aperture (f-stop), size of field and binning. However, few studies using *in vivo* fluorescent imaging to study EV biodistribution report the imaging parameters used (Garofalo et al., 2019; Tong et al., 2017a; Grange et al., 2014; Pi et al., 2018). We recommend that these important details are included in future reports of EV biodistribution.

Reported exposure times range from 1 s (Garofalo et al., 2019; Wiklander et al., 2015) to 1 min (Pi et al., 2018). Exciting the fluorophore for longer periods can increase the number of emitted photons but can also increase the emission of non-specific signals from the background or tissue autofluorescence. Similarly, excitation strength increases the amount of light that excites the fluorophore. Increasing exposure time or excitation strength can amplify the brightness of the signal. This is useful when the signal is weak, such as when the binding ratio of the fluorescent dye is low, or if the dye has a low ratio of emitted photon output to excitatory photo input, a parameter called quantum yield. However, if the experimental design is to perform an imaging time course, increasing these parameters may lead to photobleaching and an artificially smaller signal over time.

Altering the f-stop, size of field and signal binning can increase the sensitivity, enabling the detection of weak signals. The f-stop represents aperture, or the amount of light reaching the acquisition camera sensor. Unless there is a signal bright enough to cause overexposure, it is generally recommended to keep the aperture at the widest setting; this allows for a shorter exposure time, which also reduces the potential background fluorescence levels and photobleaching, and increases the signal-to-noise ratio. The camera sensor, which captures emitted photons from excited fluorophores, can be focused at different heights, which changes the field of view. Having a larger field of view means capturing more animals (*in vivo*) or organs (*ex vivo*) in one image (Fig. 1, bottom panel), simplifying comparisons between individual organs or animals. However, a large field of view means that the animals and organs are positioned further from the detection camera, and this larger distance allows for more side scatter, meaning that fewer emitted photons will be captured.

Finally, binning, or the combination of pixels detected by the camera sensor during signal processing, can also increase the sensitivity of fluorescence detection by increasing the signal-to-noise ratio. As binning combines pixels, selecting higher binning modes results in a loss of spatial resolution. This could result in reported signal areas that may extend beyond the size of the organ or tumour in which the labelled EVs localise. This could have implications when measuring the size of the area to which EVs distribute, exaggerating the area of the signal and therefore skewing the experimental results. We have found only two EV imaging studies that reported the binning settings of their image acquisition, and both used medium binning settings (Tong et al., 2017a; Grange et al., 2014).

### Imaging position of fluorescence detection

A commonly overlooked but important consideration during image acquisition is the position of the animals and organs of interest. As discussed above, *in vivo* imagers typically have the excitation light source and capture camera sensor positioned above the capture stage, to capture emitted photons from the top surface of the animal or organs imaged. For a signal to be detected, light has to be able to penetrate the tissue and excite the fluorophore to release photons. Because only a small number of these photons are released in the direction of the camera and the majority are scattered, this small number of photons needs to reach the camera without being deflected by the overlying tissue. When imaging intact animals that are not optically transparent, such as mice, it is typically assumed that only EVs near the skin can be detected, or a very strong signal is required for signal detection from intrathoracic or intraperitoneal organs. This suggests that the signal in some organs is more likely to be detected if the mouse is repositioned, e.g. kidneys are closer to the surface if the animal is placed on the capture stage in the dorsal position. Studies report imaging animals in the ventral position (Qiu et al., 2019; Haney et al., 2019; Abello et al., 2019; Wang et al., 2018), dorsal position (Zhang et al., 2018; Vinas et al., 2018; Goh et al., 2017) and on their flank (Kim et al., 2017; Bellavia et al., 2017; Ohno et al., 2013). In some cases, studies describe a mix of positions (Li et al., 2018; Zhao et al., 2016) or imaging positions were not reported. If a planned study does not define a specific organ of interest, imaging the animals in the ventral, dorsal and flank positions may increase the chance of detecting meaningful signals in the thoracic and peritoneal cavities. Similarly, removing fur and using albino animals may also improve fluorescent signal detection, as fur and skin pigments absorb and scatter photons (Pansare et al., 2012). This is particularly relevant to imaging in the far-red spectrum, where there is significant absorbance by melanin up to 650 nm in the far-red spectrum (Pansare et al., 2012).

### Autofluorescence in the gastrointestinal tract

One of the potential clearance routes of EVs is the hepatobiliary system, which deposits material into the small intestine (Choi and Lee, 2016; Wiklander et al., 2015). Therefore, the gut is an organ of interest to researchers studying EV biodistribution. When using a far-red fluorophore, it is particularly important to eliminate chlorophyll from the animal's diet to reduce autofluorescence in the gut. One study found that the autofluorescence from standard laboratory rodent food pellets was almost 10× higher when excited by light in the far-red spectrum compared to the near infrared spectrum. The same study demonstrated that switching from standard to chlorophyll-free rodent food resulted in a large reduction in the autofluorescence caused by the food pellets. Using *in vivo* imaging of mice, the authors found that removal of chlorophyll from the diet for 1-2 days with daily cage changes to prevent coprophagy were sufficient for minimising the diet-derived autofluorescence in the gut (Inoue et al., 2008). Given the large variation in brands and therefore composition of laboratory rodent chow, it may be beneficial to image untreated control animals that did not receive an injection of fluorescent EVs to determine the baseline level of autofluorescence in the gastrointestinal tract.

## Considerations for fluorescence-based biodistribution methods and analyses

*In vivo* imaging allows for tracking the change in distribution of EVs over time. However, repeated imaging of fluorescently labelled EVs will generate a weaker signal over time due to both photobleaching and natural degradation of the fluorophore, such that a decrease in detected fluorescence does not necessarily indicate clearance of EVs. By contrast, other techniques, such as radio-labelling (Varga et al., 2016), with its longer half-life or bioluminescence (Gangadaran et al., 2017; Kalimuthu et al., 2016), where fresh labelling substrate is injected immediately prior to imaging, may be better options for studying biodistribution over longer time courses. Additionally, owing to the sensitivity and resolution of fluorescence *in vivo* imaging, it is not always possible to determine the exact organs targeted in EV biodistribution. *Ex vivo* analysis, however, allows for the visualisation of organ borders without signal overlap, and therefore allows comparative quantitation. There is a mix of reported studies in which imaging was performed exclusively *in vivo* (Qiu et al., 2018; Chen et al., 2019) or exclusively *ex vivo* (Pi et al., 2018; Lee et al., 2018; Wang et al., 2017; Tong et al., 2017a), while the majority of studies paired *in vivo* imaging with *ex vivo* analysis of organs of interest (Garofalo et al., 2019; Wang et al., 2018; Hayashi et al., 2018) (Table 2).

The choice of fluorescence quantification and analysis methods depends on the experimental aims of the researcher. Most studies compare the signal intensity of the region of interest between treated and control animals. However, the accuracy of this comparison depends on the initial signal being equivalent between animals, and the successful delivery of the entire volume of labelled EVs. The majority of studies describe intravenous EV delivery by a tail vein, which is a difficult technique to master. If the tail vein injection success varies between animals (or operators) and there is variability in the partial volume of EV suspension lost in the perivascular space of the tail, the detected signal intensity may differ. Hsu et al. (2017) and Nordin et al. (2015) analysed their signals in organs as a percentage of the total signal detected, which normalises the minor differences in total fluorescence signal, enhancing comparability between groups. Additional potential variations can arise from differences in labelling efficiency between biological replicates, photobleaching during handling of the fluorescent dye or decay of fluorescence intensity due to prolonged storage of pre-labelled EVs prior to injection. Zhang et al. (2018) compared the signal intensity as a percentage of the signal of EVs prior to injection, which may mitigate some of the aforementioned variations. Including normalisation, in whichever form is most appropriate to the initial experimental design, can help reduce variation between animals within the same experimental group or between groups. Regardless of the normalisation approach, for comparability between published studies, the method for normalisation should be reported.

Another important consideration is defining relevant background signals. In addition to the aforementioned autofluorescence, most surfaces will reflect light to some degree. This can be seen in the black background of the imaging surface in *in vivo* imagers. The use of a matte, black piece of cardboard as the background may be appropriate to minimise the impact of reflective surfaces and ensure consistency over time for experiments that may span many months or years. From our experience, this background appears to be relatively consistent across different areas within the same image. However, if a comparison is being made between different imagers or if researchers are intending to combine data from multiple studies, using a black surface for a subtractive control could result in inaccuracies as the value of ‘black’ may be different across different materials, and there may be differences in the wear and tear of the material. Different tissues also have varying levels of light absorbance due to the difference in tissue composition caused by organ-specific proteins, such as haemoglobin (Zheng et al., 2010). Therefore, when subtracting background from the true signal, researchers need to carefully consider how relevant the background is for the experimental design. For example, subtracting a black background from a ‘dark’ organ, such as the liver, would yield different results from subtracting the same black background from a ‘light’ organ, such as the lungs. Several researchers have injected saline (Lai et al., 2014; Bala et al., 2015; Wiklander et al., 2015; Grange et al., 2014) as control for the background signals. Using the saline control as the baseline to subtract from the fluorescent signal corrects for any autofluorescence inherent within a particular organ. Theoretically, the result should primarily be the signal generated by the dye-conjugated EVs. This method of accounting for background signal can enhance comparability between studies.

Importantly, planning appropriate background controls together with appropriate signal normalisation can greatly reduce variation within the experiment. Experiments with large variations within groups can reduce the statistical power of the study, requiring inclusion of more animal repeats than otherwise necessary. We recommend discussion with a statistician to ensure that the experimental design is adequately powered for hypothesis testing prior to commencement of the study.

## Conclusions

*In vivo* and *ex vivo* imaging have been widely used to study the biodistribution of exogenous EVs. However, reporting on the labelling methods, excess dye removal and imaging protocols in the existing literature is not always detailed enough to allow replication of the work. This can act as a barrier for researchers wanting to conduct further studies of EV biodistribution or obtain sensitive signals to improve the accuracy of the research. This issue is further exacerbated by the lack of standardised measurements and controls.

In the future, researchers should recognise that there is no single best protocol for *in vivo* EV imaging. Instead, the protocols will likely be experiment specific, depending on the organ or area of interest in the disease model studied. For example, tracking cancer metastasis-promoting EVs should employ different tactics based on the organs in which the cancer in question preferentially metastasises, for example the skin, liver or lungs, owing to the different depths and optical properties of these organs. Regardless of the organ or disease in question, to ensure that published studies are comparable and reproducible, researchers should consider and control for the many potentially confounding variables and report the methods/parameters used in enough detail. This will foster the future growth of EV biodistribution research by improving rigor and reproducibility. With the field growing rapidly, EVs are emerging as important players in the development and progression of diseases (Box 2). For example, the use of fluorescence imaging as a tool for EV biodistribution studies has helped researchers demonstrate that EVs released from human placenta localise to the lung and liver (Tong et al., 2017a; Nguyen et al., 2021), and that this process is mediated by integrins (Nguyen et al., 2021). Additionally, although the size of the EVs released from the human placenta is increased in patients with certain obstetric conditions, such as antiphospholipid syndrome, associated with the presence of antiphospholipid antibodies (Tong et al., 2017a), this does not necessarily translate to a difference in the fate of these EVs (Tsai et al., 2022). In cancer, fluorescence imaging demonstrated that tumour cell EVs localise to future metastatic sites (Hoshino et al., 2015; Garofalo et al., 2021). In kidney disease models, mesenchymal stem cell EVs may have therapeutic potential (Gatti et al., 2011; Grange et al., 2014), and fluorescence imaging demonstrated that these EVs are able to reach the location of tissue injury (Grange et al., 2014).

As the few select examples above indicate, improving our understanding of the biodistribution of EVs in different pathologies will, in turn, help researchers understand the functional role of EVs at the tissue, organ system and organism levels in health and disease, ensuring that their translational potential for therapeutic targeting can be reached.
